# Supplementary material for: Are carbon emissions trading and green financial instruments synergistic? -Comprehensive quantitative research based on content analysis
Source: PLoS One. 2024 Mar 7;19(3):e0298601. doi: 10.1371/journal.pone.0298601 (PMC10919601; doi:10.1371/journal.pone.0298601)
Supplement: S1 Data — (ZIP) [file pone.0298601.s001.zip › Data/地级市Z_F工作报告.zip!/╔╜╢1⁄2/═■║ú╩╨/└┤╫╘╣1⁄2╓┌║┼í╛┬φ┐╦╩2╛▌═°í┐_2015.docx]

各位代表：

　　现在，我代表威海市人民政府向大会报告工作，请予审议，并请各位政协委员和列席会议的同志提出意见。

一、2014年政府工作回顾

　　过去的一年，在省委、省政府和市委的坚强领导下，市政府紧紧依靠全市人民，主动适应经济发展新常态，扎实做好各项工作，实现了经济社会持续稳步发展。

　　（一）坚持稳中求进，经济运行总体保持平稳。全市生产总值2790.3亿元，比上年增长9.8%。粮食和主要农产品产量保持稳定，工业增加值增长11.8%，服务业增加值增长10.6%。固定资产投资增长15.9%，社会消费品零售总额增长12.9%，外贸出口增长6.3%。居民消费价格上涨2.4%。一般公共预算收入220.8亿元，增长13.1%。税收占一般公共预算收入比重达到86.1%，位居全省第二。

　　（二）强化创新驱动，经济结构调整出现积极变化。设立科技孵化器扶持发展资金、科技成果转化先导资金，建成科技创新综合服务平台、技术交易市场，新增科技孵化器12家、孵化面积27万平方米，发明专利申请量、授权量分别增长36.4%和25.5%。战略性新兴产业增速明显加快，高新技术产业产值比重达到37.7%，提高1.1个百分点。推广农业实用技术，“三品一标”产品产地认定面积比重达到54.1%。建成海洋科技产业项目孵化基地，入选全国首批海洋高技术产业基地试点城市，荣成海洋生物产业园成为省级高新区。新增47艘专业远洋渔船，被确定为中国远洋水产品加工物流基地和中国海洋食品名城。成功创建全国旅游标准化示范城市，新增4A级景区3家，新建6处国家级休闲渔业示范基地。加快发展新兴服务业，电子商务交易额增长38.9%，信息消费增长20%。三次产业比重由8：51.5：40.5调整为7.7：50.5：41.8。

　　（三）统筹区域发展，基础设施建设取得新突破。东部滨海新城开发建设全面展开，重点区域开发建设加快推进。文登撤市设区，进入同城化发展的新阶段。青荣城际铁路正式通车，威海北站综合交通枢纽、301省道外移、威海机场升级改造等工程完工投入使用，荣文高速公路、文莱高速公路加快建设。蓝色经济区建设16个重大事项进展顺利，国家海产品质检中心一期工程完工，海洋商品国际交易中心投入运营。组织实施142项市级重点项目、67项重点工业技改项目和36项城建重点工程。新建农村新型社区25个，实施棚户区改造2.2万户，补助建设改造农房1.5万户，入选国家新型城镇化综合试点城市。深入推进智慧城市建设，开通智能公交服务，50条公交线路、520辆公交车实现智能调度和到站信息手机实时查询；完成智慧交通一期工程，实现道路交通智能化指挥调度;成为首批信息惠民国家试点城市、宽带中国示范城市。

　　（四）优化营商环境，市场主体活力有效激发。推行建设工程项目模块化审批，实施市场主体设立联审联批，取消下放行政审批事项175项，市级审批项目实现能取消下放的全部取消下放。全市新登记各类市场主体3.97万户，增加69.6%，其中新登记私营企业9956户，增加106.6%，创历史新高。出台支持企业创新发展的各类政策，设立小微企业贷款风险补偿基金，扩大科技支行扶持范围，引进8家金融机构，29家企业实现场外市场挂牌，其中新三板10家；社会融资总量达到420.3亿元，增长33.3%。成立市小微企业创业服务中心，创办威海创业大学，新建大学生创业孵化基地4处；发放创业小额担保贷款2.3亿元，扶持2336人自主创业。引进各类人才3.1万人，新增高技能人才6834人，6个创新团队入选泰山学者蓝色产业计划。

　　（五）深化改革开放，制度创新活力加速释放。积极争创改革试点带动相关领域改革，新增10个国家级、省级试点。转变国有资本经营管理模式，成立国有资本运营公司。建立并实施政府向社会力量购买服务制度。实施中小学校长职级制改革试点、初中学业考试、责任督学挂牌督导等教育领域改革，成为全国义务教育学校管理标准化实验区。完成县级公立医院综合改革。农村三项改革扎实推进。全面实施关检合作“三个一”口岸通关模式。加快建设韩国食品日用品集散地。实际到账外资10.1亿美元，增长10%；实际利用内资638亿元，增长15.2%。企业走出去步伐加快，备案核准境外投资4.2亿美元，增长51.5%；对外承包工程营业额达到7.5亿美元，增长40.1%。

　　（六）坚持生态立市，生态环境保护力度持续加大。深入开展大气污染防治行动，加强扬尘污染、弃土堆清理、散流物体运输、燃煤小锅炉专项整治，建成燃煤机组脱硝设施22处，淘汰黄标车及老旧车5.7万辆，省测“蓝天白云”天数达到326天，空气质量优良率保持全省首位。实施6个污水处理厂提标改造工程，治理河道63公里，完成80座病险塘坝除险加固工程。实施15个海岸带修复整治项目。处置闲置土地、盘活低效利用土地1.5万亩，关停采石场26家。完成20个旧生活区改造和255个村的环境综合整治，成片造林12万亩。万元生产总值能耗预计下降4.5%，主要污染物排放量持续下降。

　　（七）强化民生保障，公共服务水平进一步提升。各项民生保障政策加速并轨整合，实施城乡统一的居民基本医疗保险制度，将外来务工贫困家庭和支出型贫困家庭纳入最低生活保障，独生子女父母养老补助实现城乡全覆盖。上调了居民基本养老保险基础养老金、企业退休人员基本养老金、失业保险金、城乡最低生活保障、农村五保、高龄津贴、残疾人护理补贴等标准，提高了居民医保、基本公共卫生服务的财政补助标准。扎实办理25项民生实事，开工建设公共租赁住房1201套；新增节能环保型公交车290辆；新增机构养老床位4547张；建成市乒羽健身中心，新建城市健身慢行道32.5公里；新建公办幼儿园14所，为41所学校建设塑胶跑道和运动场地，市中小学生综合实践教育中心投入使用，农村中小学新模式校车实现全覆盖。新增城镇就业4.5万人，转移农村劳动力1.9万人，城镇登记失业率降为1.52%。城镇居民人均可支配收入34254元，农民人均纯收入17296元，分别增长8.9%和11%。

　　（八）加强文化建设，文明创建工作取得新成效。扎实推进全国文明城市创建工作，突出全民共建、全民共享的理念，结合创建工作办理了改善生活环境、治理背街小巷、升级便民设施、规范经营秩序等关系群众切身利益的身边事20余万件，使创城的过程成为改善城市管理、为民办实事的过程。在中央文明委刚刚公布的公示通知中，威海市进入全国文明城市候选名单，测评成绩位列地级市第一名。广泛开展社会主义核心价值观宣传教育活动，被评为全省“四德”工程建设示范市。开展幸福威海文化活动，成为全省首批公共文化服务体系示范区。开展纪念甲午海战120周年系列活动，组织仁川亚运会火炬传递活动，举办长距离铁人三项世界锦标赛，提升了城市影响力。

　　（九）创新社会治理，社会保持和谐稳定。深化海陆一体平安建设，采取信息化手段强化社会治安综合治理，社会治安群众满意度位居全省第一。理顺食品药品监管体制，启动国家食品安全城市创建工作，成为全国肉菜流通追溯体系建设试点城市。启用安全生产网格化监管信息平台，采取政府购买服务方式引入专业力量参与安全生产检查，各类生产安全事故下降42.9%。国防动员、人民防空、民兵预备役工作进一步加强。各项社会事业都取得新进步，被评为全国“六五”普法中期先进城市。

　　（十）坚持依法行政，政府自身建设不断加强。扎实开展党的群众路线教育实践活动，机关作风进一步改进。完成新一轮政府机构改革，组建政府法律顾问团，扩大政府信息公开范围，健全行政复议工作运行机制。开通市民网，增强了网上办事功能。整合88条公共服务热线，建立政务服务、应急联动、便民服务3个热线平台。自觉接受人大的法律监督、政协的民主监督，办理人大代表建议和政协委员提案240件。坚持和完善民主决策机制，广泛征求群众意见建议。强化监察、审计监督，开展廉政风险防控“回头看”活动，加强防止利益冲突试点工作，法治政府、服务型政府、廉洁政府建设取得新成效。

　　各位代表，政府工作取得的每一份成绩，都得益于方方面面的理解和支持。在此，我代表市政府，向全市人民，向人大代表和政协委员，向各民主党派、工商联、各人民团体和各界人士，向驻威人民解放军、武警官兵，向各位老领导、老同志，向所有关心支持威海发展的海内外朋友，致以崇高敬意和衷心感谢！

　　各位代表，去年5月29日，我市里口山发生山火，暴露出基层森林防火工作措施不到位、基础设施不完善等问题，我们必须深刻反思，认真吸取教训，采取有力措施，全力保障人民群众的生命财产安全。在山火扑救过程中，威海人民表现出来的文明素质和精神风范令人感动，各级各部门快速反应、果断处置，部队官兵和专业队伍奋不顾身、冲在第一线，广大干部群众众志成城，每一个热爱家园的威海人，都以各种方式奉献爱心、贡献力量，迸发出空前的凝聚力，汇聚起强大的正能量。这种宝贵的精神财富，也是激励我们不断做好工作的力量。

　　我们还清醒地认识到，我市经济社会发展仍存在不少矛盾和问题。经济下行压力较大，造船等行业很不景气，部分企业生产经营困难，我们引进和培植的新项目、大项目、好项目还不够多，产业领域的有效投资不足，经济稳定增长的基础还不牢固。推进经济结构调整仍是我们需要解决的重点难题，服务业增加值比重低于全省平均水平0.9个百分点，制造业发展层次和水平有待提升，开放型经济也面临转型升级难题。全社会科技创新投入不足，人才瓶颈制约依然突出。一些园区和镇街的公共服务设施不够完善，市域一体化的公共服务体系需要加强。全市还有120个省定贫困村，部分群众的生活还比较困难。安全生产事故时有发生，基层基础工作还不牢固。部分政府工作人员法治意识淡薄，作风转变不到位，企业和群众反映的突出问题没有得到根治，廉政建设还需要进一步加强。我们将按照逆向切入的思路，采取有效措施，扎实解决这些问题，努力加强和改进政府工作。

二、2015年政府工作目标和总体思路

　　各位代表！认识新常态，适应新常态，引领新常态，是当前和今后一个时期我国经济发展的大逻辑。习近平总书记视察山东重要讲话和重要批示精神，是我们在新常态下做好各项工作的根本遵循。2015年政府工作的总体要求是：全面贯彻党的十八大和十八届三中、四中全会精神，以邓小平理论、“三个代表”重要思想、科学发展观为指导，深入贯彻习近平总书记系列重要讲话精神，坚持稳中求进工作总基调，坚持以提高经济发展质量和效益为中心，主动适应经济发展新常态，坚定推动工作指导重大转变，努力在转方式调结构、强化创新驱动方面有新突破，在深化改革扩大开放、激发市场活力方面有新成效，在扩大内需、释放发展潜力方面有新举措，在加强社会治理、弘扬优秀传统文化方面有新进展，深入实施生态立市、科教兴市、产业强市战略，推动全域城市化、市域一体化发展，着力保障改善民生，促进经济平稳健康发展和社会和谐稳定，加快建设现代化幸福威海。

　　主要预期目标为：生产总值增长8.5%左右，一般公共预算收入增长10%左右；固定资产投资增长15%左右，社会消费品零售总额增长12.5%左右，居民消费价格涨幅控制在3%左右；新增城镇就业3.3万人，转移农村劳动力1.1万人，城镇登记失业率控制在2%以内；外贸进出口总额增长5%左右，实际到账外资稳步增加，实际利用内资增长15%左右；城镇和农村居民人均可支配收入分别增长9%左右和10%左右。全面完成省下达的年度节能减排约束性目标。

　　实现上述目标，要深刻把握经济发展新常态的内涵和规律，打破传统思维惯性束缚，推动工作指导重大转变。一是坚持以提高经济发展质量和效益为中心。切实把经济工作的着力点放到转方式调结构上来，下大力气突破制约我市经济转型升级的瓶颈问题，着力在提质增效升级上取得新突破。二是坚持走创新驱动发展的道路。彻底摒弃依靠资源环境消耗的增长方式，更多依靠人力资本质量和技术进步增强发展动力，更多依靠新技术、新产品、新业态、新商业模式提升产业层次，着力让创新成为驱动发展新引擎。三是坚持向深化改革开放要动力。以先行先试的胆识和魄力，深入推进制度创新持续释放改革红利，深度融入国家开放战略抢抓发展机遇，着力增创对外开放新优势。四是坚持用法治思维和法治方式开展工作。牢固树立依法行政的理念，依法履行政府职能，依法约束行政权力，依法规范行政行为，在法治轨道上深化改革、推动发展、化解矛盾、维护稳定。五是坚持把人民对美好生活的向往作为奋斗目标。巩固党的群众路线教育实践活动成果，把一切为了群众、一切依靠群众作为工作的出发点和落脚点，凝聚方方面面的智慧和力量，形成干事创业的强大合力，不断开创各项工作新局面。

三、2015年政府工作安排

　　（一）加快推进经济转型升级。坚持产业强市、工业带动，按照一产抓特色促跨越、二三产抓结构上规模的思路，推动传统产业向中高端迈进，增强现代制造业和新兴服务业的支撑作用。

　　努力保持经济稳定增长。注重发挥投资的关键作用、消费的基础作用、出口的支撑作用，支持企业扩大投资、开拓市场，有效应对经济下行压力。组织实施155项市级重点项目、110项重点工业投资项目、42项城建重点工程，以更大的力度支持内涵效益型投资，鼓励更多的社会资本参与公共基础设施建设，进入公共服务领域。深入实施消费惠民、消费便民工程，鼓励发展网络消费等新型消费方式，打造多点支撑的消费增长格局，力争信息消费规模超过150亿元。注重发挥企业家才能，尊重企业家、理解企业家、培育企业家、服务企业家，建立更加有效的对接服务机制，提振企业家发展信心，更好地发挥企业家作用，夯实经济增长微观基础。

　　培育发展新型制造业。实施产业集群“3+4”发展战略，大力发展新信息、新医药、新材料等战略性新兴产业，促进传统产业加速向产业链两端延伸，重点突破医疗器械产业、海洋生物产业集群发展。继续化解过剩产能、淘汰落后产能，促进造船等行业转型发展。促进信息技术与传统制造业跨界融合，推广应用云计算、大数据、物联网等信息技术，培植机器人、3D打印产业。降低技术改造贴息门槛，支持企业提升技术装备水平。加快建设医疗器械检验检测中心等公共检测平台，推广应用工业设计云平台。加快军民结合产业发展。引导企业加强质量和标准化体系建设，发展自主品牌。

　　鼓励发展新兴服务业。着眼于拉长服务业“短腿”，今年把促进服务业发展提速、比重提高、水平提升作为一项重大课题来研究，运用“敲开核桃”的方式，分行业解剖分析，制定转型升级实施方案。鼓励发展互联网经济，推动工业企业开展电子商务应用，支持环翠区争创国家电子商务示范基地，力争电子商务交易额突破1000 亿元。加大金融机构引进力度，促进金融业持续健康发展。加强现代物流园区规划布局，支持发展第三方物流。培育发展法律、咨询、会计、审计等商务服务业。鼓励社会资本进入教育、医疗、健康、养老、文化、体育等公共服务业。引导企业分离和外包非核心业务。服务业增加值比重提高1个百分点。

　　大力发展高效生态安全农业。加快转变农业发展方式，推广现代农业技术，稳定粮食和主要农产品产量。支持发展新型农业经营主体，新培育家庭农场100个、农民专业合作示范社50个。推动本地农产品品牌化建设，新增农业标准化生产基地8万亩，争创国家农产品质量安全市。加快建设供销社为农服务中心，畅通农产品流通渠道。提升苹果、西洋参、无花果、茶叶、苗木花卉等特色农业发展层次，促进一产向二产三产跨越延伸。

　　积极发展海洋特色产业。深入推进蓝色经济区建设，着力培植10个海洋经济特色园区，支持荣成海洋高新技术产业园优化专业服务，提升产业层次。设立现代海洋渔业发展专项资金，促进海洋渔业转型升级。加快海外综合性远洋渔业基地建设，加强涉外渔船管理。支持建设综合性海洋生物遗传育种中心，突破水产苗种产业。依托海洋商品国际交易中心，做大海产品交易市场。积极开发海洋生物新材料、海洋生物保健品、新型海洋药物、海洋冷冻调理食品等新产品，提高海产品增值水平。推进国家浅海海上综合试验场建设，打造海洋仪器装备研发制造基地。

　　（二）全面优化创新创业环境。进一步简政放权，优化行政服务，营造有利于大众创业、万众创新的政策环境和制度环境。

　　优化行政服务环境。下大力气拆除不必要的审批门槛，去除不应有的繁文缛节，铲除权力寻租的土壤。全面推行市场主体设立联审联批、建设工程项目模块化审批，切实减少审批环节，避免部门之间推诿扯皮。最大限度精简前置审批，推动事前审批向事中事后监管转变，从制度上解决管得过多、管得过死的问题。完善市县镇一体的电子审批系统，丰富市民网服务功能，扩大网上办事范围，以接地气的办事流程、快精准的办事效率，进一步方便群众办事。加强与行政审批相关中介机构监管，排查清理设定强制性中介服务、指定中介机构和中介机构垄断定价等问题，在市级权限内引进更多中介机构打破垄断，清除隐性审批行为。加强水电气暖、通信、广电网络等公共服务行业监管，切实优化公共服务。规范涉企执法检查、收费行为，严肃查处吃拿卡要等痼疾，着力解决不作为、乱作为等问题。以更大的决心清除“灰色地带”，打破“潜规则”，打碎“玻璃门”、“弹簧门”，以企业和群众的真实感受来检验简政放权的实际成效。

　　优化创新支撑环境。实施“一产业一特色”科技创新行动计划，推进创新型城市建设。组织实施“千帆计划”，培育科技型中小企业1000家。推动科技金融结合，引导企业加大研发投入。支持建设机器人与智能装备产业研究院、中韩生命科技研究院、海洋药物与功能食品研究院等高水平的研发机构和产业技术创新联盟，促进产学研协同创新。整合科研基础资源，构建统一检测服务联盟和仪器设备租赁平台。发挥科技创新综合服务平台作用，健全科技标准化评价体系，鼓励发展科技中介服务，繁荣技术市场。促进各类开发区完善公共创新服务平台，提升产业项目承载力。完善创业孵化链条，推动有条件的企业创办各类专业孵化器，市级以上科技孵化器发展到20家，新增孵化面积20万平方米。促进专利创造与运营，发明专利申请量、授权量均增长20%以上。

　　优化创业和就业服务环境。完善创业服务体系，进一步降低创业门槛和创业成本。健全创业培训实训机制，积极创建省级示范创业大学，全年完成创业实训3000人。鼓励社会力量创办创业孵化基地，完善各类创业孵化平台功能。实施万名大学生聚威创业计划，引领扶持大学生自主创业。继续开展“快易贷”行动，积极引入风险投资机构，为创业者提供资金支持。实施城乡统一的就业失业登记制度和就业扶持政策。拓宽高校毕业生就业渠道，实施农民工职业技能提升、权益保障、公共服务行动计划，促进就业困难群体充分就业。规范用工中介市场，依法取缔黑中介。严厉打击恶意欠薪等违法行为，构建和谐劳动关系。

　　优化人才发展环境。深入实施“英才计划”，大力强化招才引智，在全社会营造尊重知识、尊重人才的氛围。实行重点产业和新兴产业人才需求目录发布制度，支持企业引进紧缺急需的专业人才。实施企业家培育计划，提升企业经营管理队伍的职业化水平。实施海外人才聚集、企业博士聚集计划，打通人才交流快速通道，吸引高端人才携带项目和科技成果来我市创业。实施首席技师、乡村之星选拔计划，加强实用型人才队伍建设。实施文化名家、教育名家、首席专家等计划，在文化、教育、卫生等公共服务领域培养一批拔尖人才。完善人才激励机制，营造吸引人、留住人、激励人、成就人的良好人才环境。全年引进各类人才2万人以上，培养高技能人才5000人以上。

　　（三）全力增创开放新优势。中韩自贸协定将正式签署实施，威海作为中韩经贸文化交流“桥头堡”的地位更加突出。我们要紧紧抓住与仁川自由经济区合作的有利机遇，借力提升开放型经济发展水平，谋求对外开放新突破。

　　在更高层次上推进对韩交流合作。加强战略谋划和对策研究，在贸易、投资、服务、产业合作等领域，积极争取先行先试政策，扎实推进中韩（威海）经济合作示范区建设，努力实现对韩交流合作新突破。推动旅游业合作创新试点，打造“威韩连线”旅游品牌。创新中韩陆海联运业务模式，加快推动中韩港铁联运。推进韩国食品日用品集散地建设，依托威海国际物流园，打造韩国商品中国分拨中心。推动农产品质量认证互信互认，促进中韩共建食品农产品质量安全示范区。规划建设中韩信息技术产业园。加大韩国金融机构引进力度，探索在双方优势互补产业设立产业基金。

　　提高通关便利化水平。加强与仁川口岸互联互通，推进中韩优秀进出口企业互认工作，让企业享受更多通关便利。加快口岸开放步伐，稳步推进进口冰鲜水产品口岸建设，争取获批进境肉类指定口岸。推动内销选择性征税、集中汇总纳税、保税展示交易等海关监管制度创新，实现口岸通关全流程电子化管理。抓好综合保税区申报建设。

　　提升招商引资实效。深化招商工作机制创新，建立招商培训平台，推行专业化招商、产业集群招商和产业链招商。强化以商招商，鼓励外资企业以技术出资、境外借款、利润再投资等方式增资。鼓励国内外企业采取并购、风险投资等方式加强投资合作。发挥各类对外交流资源优势，拓宽招商引资渠道。加大央企和民营企业招商力度，促进国内经济合作。

　　扩大对外经贸合作。支持新型贸易业态发展，开展跨境电子商务业务。大力发展服务贸易，加快培育服务外包产业。培育自主出口品牌，引导加工贸易企业转型升级。支持内贸企业拓展对外贸易功能，促进内外贸一体化发展。实施进口促进战略，扩大大宗商品、先进装备进口规模。加强与国家海外投资基金合作，支持企业安全高效走出去，到境外建立生产基地、营销网络和研发中心，提高对外投资效率和质量，拓展对外合作新空间。

　　（四）积极推动市域一体化发展。强化重点区域带动，加快新型城镇化建设步伐，统筹城乡区域协调发展。

　　推动重点区域开发建设。实施四个“三年突破”战略，加快东部滨海新城建设，推进文登区与中心城区对接融合、同城化发展，促进南海新区提升集聚发展能力，加快乳山市产业结构调整。推动高区、经区、临港区三个国家级开发区上档升级，提升各类省级开发区发展层次，增强公共服务能力，促进各类开发区切实发挥创新发展的引擎作用。强化产业招商和项目建设，加快双岛湾科技城、好运角旅游度假区、乳山滨海新区、里口山风景名胜区等重点区域开发建设进程。

　　统筹城乡基础设施建设。扎实推进城际铁路威海站综合交通枢纽、荣文高速公路、文莱高速公路、香水河大桥等重点交通设施项目。实施25个电网建设项目和12项燃气热力工程。完成刘公岛客运中心搬迁，并建成市级旅游公共服务中心；依托4个城铁站配建旅游集散中心，优化旅游服务功能。推进休闲植物园、里口山城市山地公园、城市绿道等工程建设。完成棚户区改造8000户。实施既有居住建筑供热计量及节能改造948.3万平方米。新建农村新型社区24个。实施病险塘坝除险加固、农村规模化供水工程，改造农村公路150公里。完成39个旧生活区、231个村的环境综合整治，优化城乡居住环境。

　　加强城市规划与管理。做好“十三五”规划编制工作，科学编制城乡规划、新型城市化发展规划、城市环境总体规划，强化各类规划的统筹衔接。加强智慧城市建设，推进智慧旅游、智慧交通续建项目，实施智慧环保、智慧教育、智慧医疗等项目。发展绿色公交、便民公交、智能公交，新增280辆节能环保型公交车，新开、优化公交线路7条，新建电子站牌100套。优化城市精细化管理的办法和流程，扩容改造城市综合管理信息系统，将文登区、临港区纳入城市精细化管理范围。实施城市生命线智能工程，提高城市基础设施管理智能化水平。

　　（五）深入推进重点领域改革。坚持以改革创新精神推动各项工作，最大限度激发市场和社会活力，增强发展新动能。

　　深化财税管理改革。健全全口径政府预算体系，设立预算稳定调节基金，实现跨年度预算平衡。创新财政支持产业发展方式，紧跟全市产业发展需要，设立分行业的产业投资基金，推进种子基金、天使基金、创业投资基金发展。健全债务风险预警及化解机制，加强政府性债务管理，规范举债行为，控制举债规模。

　　推进金融改革创新。大力发展普惠金融，推动金融机构创新金融产品和服务模式，加大服务中小微企业和“三农”力度。扩大科技支行覆盖范围，加大对科技型中小企业信贷支持。鼓励金融机构创设服务海洋经济发展的特色金融产品。推进企业上市和科技型企业场外市场挂牌，拓宽直接融资渠道。争取民营银行试点。促进小额贷款公司、融资性担保公司、民间资本管理公司规范健康发展。鼓励保险公司开发新型险种，拓宽利用保险资金的途径。加强金融监管，防范区域性、系统性金融风险。

　　完善公共资源管理机制。加强国有资本运营管理，构建国有资本流动重组、布局调整的有效平台。建立出资人权利清单制度，深化国有资本经营预算管理。大力发展混合所有制经济，推动机关事业单位所办企业脱钩。促进海域资源使用市场化配置，完善海域使用权收回补偿制度，规范和盘活海域流转二级市场。

　　健全城乡一体化体制机制。开展国家新型城镇化综合试点工作，争创全国中小城市综合改革试点。统筹推进示范镇建设。基本完成农村土地承包经营权确权登记。健全农村集体“三资”监管制度，完成中心城区城中村集体产权制度改革。探索开展农村产权交易市场建设试点工作。

　　深化行政管理体制改革。推进事业单位分类改革，全面推开事业单位绩效考核。稳步推行县以下机关公务员职务与职级并行制度。探索跨部门、跨行业综合执法试点工作，理顺城管执法体制，合理配置执法力量，减少执法层级。

　　（六）着力加强生态文明建设。坚持生态立市，强化“三生”共融理念，健全生态建设与保护长效机制，持续改善环境质量，巩固生态环境比较优势。

　　加强污染综合治理。深入开展大气污染防治行动，强化扬尘、异味、废气等污染治理，严格落实散流物体密闭运输、黄标车区域限行措施，进一步提高空气质量。深入推进水生态环境治理，加强水功能区和排污企业监管。强化农业面源污染防治，加强土壤环境保护工作。启动市区养殖用海三年整治行动。加强噪声污染治理。建成餐厨废弃物处理和利用设施。加快推进智能化环境执法监控平台建设，新建6个空气质量和10个水源地水质自动监测站。

　　加强资源节约利用。实施重点工业企业清洁生产提升计划，大力发展循环经济。严格落实节能评估审查制度，积极推行合同能源管理，加强建筑节能工作。实行最严格的水资源管理制度，加大饮用水水源地保护力度，加强地下水和地热水资源管理。扎实推进增减挂钩和工矿废弃地复垦调整利用工作，加大闲置和低效利用土地处置力度，提高土地投资强度和节约集约利用标准。

　　加强生态环境建设。实施生态红线管理，建立生态补偿机制和生态环境损害责任追究制度。强化山体、湿地、水系、沙滩、海域保护，实施29个生态修复项目，加强刘公岛、小石岛、海西头等7处国家级海洋特别保护区建设。实施森林防火能力建设三年行动计划，加快完善森林防火基础设施，成片造林8.8万亩。

　　各位代表！优美的生态环境是威海最宝贵的财富，保护生态环境是我们义不容辞的责任。今年集中力量抓好八个重点事项，全面完成热电企业脱硝设施建设，确保重点企业脱硫脱硝设施全部正常运行；试点推行燃煤机组提标改造，达到燃气机组排放水平，实现超低排放；拆除58座分散燃煤锅炉；淘汰3.9万辆黄标车和老旧车，全面完成黄标车淘汰任务；全面完成330座加油站的油气回收治理工程，严厉打击销售劣质成品油的违法行为；实施3条农村中小河道和13条城郊河道综合治理；推进6座污水处理厂提标改造；完成132家规模化畜禽养殖场污染整治，努力完成“十二五”节能减排约束性目标任务，把威海建设成山清水秀、宜居宜业的美丽家园。

　　（七）大力加强文化建设。威海有着深厚的文化底蕴，秦汉文化、道教文化、民俗文化、海权文化、红色文化与现代文明交融，形成特色鲜明的威海文化，是齐鲁文化的重要组成部分。我们要对这些历史文化资源进行挖掘和阐发，大力弘扬优秀传统文化，加快推进文化建设，提升城市文化软实力。

　　积极践行社会主义核心价值观。深入开展群众性精神文明创建活动，强化未成年人思想道德教育，推进美德威海建设，弘扬传统美德，倡树新风正气。加强诚信威海建设，大力开展公众诚信宣传教育，探索建立公共联合征信系统，统筹推进政务诚信、商务诚信、社会诚信、司法公信建设。加强爱国主义教育、民族团结教育，举办好纪念抗日战争暨世界反法西斯战争胜利70周年活动。

　　加强文化遗产保护利用。开展本土文化资源普查，鼓励本土历史文化课题研究，组织实施“乡村记忆”工程，延续城市历史文脉。加强老洋房、海草房等文化遗产保护利用。推动第二轮地方志编修。加强档案信息资源挖掘利用，开展抗日战争档案公布活动。加强尼山书院建设，开展经典诵读活动。

　　完善公共文化服务体系。建设公共文化服务云平台，推进公共文化服务标准化建设。加快建设市图书馆、群众艺术馆、档案馆，改造威海剧院。办好文化惠民实事，开展幸福威海文化活动。扎实推进全民阅读活动，完善提升社区图书室、农家书屋功能，扩大公共图书资源覆盖范围。

　　推动经济文化融合发展。大力发展文化创意产业，建设一批具有较强竞争力的文化产业示范园区、骨干文化企业和文化品牌。发挥文化产业融资战略合作联盟作用，探索建立向文化实体提供融资担保新机制。促进文化旅游融合发展，开发高铁旅游市场，推进休闲渔业和乡村旅游业特色发展。大力发展体育产业，促进体育消费，举办长距离铁人三项世界系列赛。办好第八届中国威海国际人居节及国际建筑设计大奖赛。

　　（八）切实保障和改善民生。强化政府责任，更加注重保障基本民生，更加关注低收入群众生活，更加重视社会大局稳定，编密织牢保障民生安全网。

　　办好人民满意教育。深化教育综合改革，促进各类教育协调发展。大力发展素质教育，推动义务教育优质均衡发展。实施中小学办学条件标准化提升工程，改善133所中小学校的办学条件。实施第二期学前教育行动计划，新建、改扩建10所公办幼儿园。实施体卫艺教育三年行动计划，开展足球进校园等活动，为38所学校建设塑胶跑道和运动场地。扎实推进普通高中学业水平考试和综合素质评价改革。加快建设适应经济社会发展需要的现代职业教育体系，扩大中等职业教育“双证互通”试点范围，开工建设威海中等职业学校。推进中小学校长职级制改革试点工作，探索建立“县管校聘”教师管理体制。促进民办教育规范办学，探索建立非营利性民办学校教师与公办学校教师同等待遇的社会保障机制。强化与驻威高校共建，推进威海职业学院校企合作示范基地建设，实施山东药品食品职业学院扩建工程，推进北京交通大学威海校区、山东交通学院威海校区的建设与招生工作。

　　提高社会保障水平。实施机关事业单位工作人员养老保险制度改革，继续提高企业退休人员基本养老金。健全居民大病保险制度，扩大基本医疗保险定额结算病种范围，居民医保财政补助标准由每人每年330元提高到390元。启动人力资源和社会保障公共服务一体化信息便民工程建设，新增自助服务终端600台，实现各项业务市域通收通办。整合社会救助资源，建立社会救助申请综合服务平台，切实发挥社会救助托底功能。实施残疾人奔小康工程，改建市残疾人康复中心。推广医养融合发展模式，大力发展养老服务业，新增机构养老床位1300张。对50个贫困村实施精准扶贫，改善贫困户的生产生活条件。扩大住房公积金保障范围。强化保障性住房建设，新建公共租赁住房3287套。

　　强化医疗健康服务。促进基本公共卫生服务均等化，基本公共卫生服务财政补助标准由人均35元提高到40元。免费发放信息卡，实行新生儿预防接种信息化管理。提高“两癌”筛查财政补助标准。设立疾病应急救助基金，加强医疗应急保障工作。加快推进市全科医生培训基地、文登中心医院综合病房楼等重点项目建设，新建改造农村急救站23处、农村标准化卫生室142处。推进中医药综合改革，扩大中医药优势病种收费方式改革实施范围，积极创建省级中西医结合区域医疗中心。建立医疗专家到社区医疗机构巡诊制度，提升基层医疗服务能力。加强计划生育特殊困难家庭救助。

　　加大社会治理创新力度。深化海陆一体平安建设，健全立体化社会治安防控体系。加快公共安全视频监控系统建设，提升社会治安管控能力。全面推行安全生产网格化监管，健全隐患排查治理和安全预防控制体系。积极创建国家食品安全城市，建立肉菜流通追溯管理系统，严厉打击食品安全违法行为。强化应急教育培训和预案演练，提高突发事件的应对处置能力。加强法律援助、司法救助，完善人民调解与行政调解、司法调解衔接联动工作机制，提升基层化解矛盾纠纷能力。

　　协调发展各类社会事业。广泛开展全民健身活动，新建公共体育场地和综合性健身设施7处，新增体育设施向社会开放学校12处，组织全民健身运动会等36项群众性体育赛事。强化基层民主法治建设，健全基层群众自治组织。支持社会组织孵化园发展，发挥社会工作者的作用，构建社区公共服务、便民利民服务、志愿互助服务相互衔接的社区服务体系。加强国防动员、民兵预备役、人民防空和打击走私等工作，抓好双拥共建和优抚安置。加强民族宗教、妇女儿童、仲裁、气象、防震减灾和对口支援等工作。

　　各位代表！按照群众的需求和意愿，今年我们集中力量办理26项民生实事。各项民生实事实行重点督办，办理事项向群众公开承诺，办理过程接受群众监督，办理结果接受群众评议，努力把实事办实、好事办好。

四、大力加强法治政府建设

　　各位代表！建设法治政府、服务型政府和廉洁政府，是本届政府的不懈追求。我们一定牢固树立宗旨意识，始终敬畏宪法和法律，大力推进依法行政，在法治轨道上开展各项工作。

　　依法履行政府职能。坚持法定职责必须为、法无授权不可为，督促政府工作人员带头学法尊法守法用法，严格按照法定权限和程序行使权力、履行职责。依法向社会公开行政权力清单、政府责任清单，固化行政权力行使程序。完善政府重大行政决策程序，全面推行政府法律顾问制度，健全合法性审查和风险评估机制。完善规范性文件制定程序，防止部门利益法制化。加强行政执法监督，建立重大执法决定事前法制审核、事后备案制度和执法全程记录制度，公开行政处罚裁量基准，避免执法随意性。加强行政违法预防工作，优化行政复议工作机制。强化法无例外、政府必须守法的理念，今年各级政府和所属单位都要带头清偿拖欠的工程款，补交应缴税费。

　　依法接受各方面监督。自觉接受人大的法律监督、政协的民主监督、媒体的舆论监督、群众的社会监督，认真办理人大代表建议和政协委员提案。加大政府信息公开力度，拓宽政务公开范围，依法公开公共资源配置、重大建设项目批准和实施、社会公益事业建设等领域的政府信息，今年市县两级政府、部门和“三公”经费预决算全面公开，保障群众知情权、监督权。完善新闻发言人制度，办好政府网站和政务微博、微信，及时回应社会关切。强化监察、审计等内部监督，行政事业单位和国有企业都要按计划进行全面严格的审计，行政事业单位签订合同及合同履行情况都要按规定报告，努力把规矩定得细一点，制度执行得严一点，提高人民群众的满意度和信任度。

　　加强政府廉政建设。坚持党风廉政建设与经济社会发展一起谋划、一起部署、一起考核，强化“一岗双责”，认真落实党风廉政建设责任制。认真遵守政治纪律和政治规矩，严格落实中央八项规定精神和省委、市委实施办法，始终保持反“四风”高压态势。牢固树立过“紧日子”思想，严格执行厉行节约反对浪费各项规定，深化公务用车制度改革，严控“三公”经费支出，从细节抓起，防微杜渐。持续开展廉政警示教育活动，加强廉政风险防控和防止利益冲突工作，行政权力集中的部门和岗位必须分级授权、定期轮岗，发生过违纪违法案件的岗位必须进行流程再造、弥补监管漏洞。严肃查处各类违纪违法案件，保持惩治腐败高压态势，以为民务实清廉的政府形象，赢得人民群众的信任和支持。

　　各位代表！梦想承载希望，信念凝聚力量，人民群众的期待始终是我们奋斗的目标和做好工作的动力。让我们紧密团结在以习近平同志为总书记的党中央周围，在市委的坚强领导下，凝心聚力，真抓实干，为加快建设现代化幸福威海而努力奋斗！
